# Influences and Failure Analysis of the Interaction Between Melt and Gas on Double-Layer Gas-Assisted Extrusion Molding of Polymer Micro-Catheters

**DOI:** 10.3390/polym17040504

**Published:** 2025-02-15

**Authors:** Zhong Ren, Xiaozhen Deng, Haibo Ji

**Affiliations:** 1 Jiangxi Provincial Key Laboratory of Advanced Electronic Materials and Devices, Jiangxi Science and Technology Normal University, Nanchang 330038, China; 2 Nanchang Key Laboratory of Optic-Electronic Detection and Information Processing, Jiangxi Science and Technology Normal University, Nanchang 330038, China; 3 Jiangxi Provincial Key Laboratory of Precision Drive and Equipment, School of Mechanical Engineering, Nanchang Institute of Technology, Nanchang 330099, China; jhb18738203146@163.com

**Keywords:** polymer micro-catheters, double-layer gas-assisted extrusion molding, failure analysis, experiment study, numerical simulation

## Abstract

Although the extrudate swelling, melt fracture, and extrusion deformation of polymer micro-catheters in traditional extrusion molding can be eliminated via the double-layer gas-assisted extrusion (DL-GAE) method, some failure problems are generated under unreasonable process conditions. To ascertain the reasons for failure in DL-GAE molding of polymer micro-catheters, the influences of the interaction between the melt and double assisted gas on the DL-GAE molding of polymer micro-catheters were experimentally and numerically studied. Meanwhile, a DL-GAE die and experimental system were designed and constructed. We analyzed the influence laws of the melt and assisted gas on the DL-GAE molding of polymer micro-catheters, as well as reasons for the molding’s failure. Our studies demonstrate that under the condition of stable DL-GAE, as the melt flow rate increases, the wall thickness and diameter of polypropylene (PP) micro-catheters increase. When the melt flow rate continuously increases, the stability of the assisted gas is destroyed, resulting in the failure of DL-GAE. In addition, under synchronized pressures of a double gas-assisted layer, the diameters of the micro-catheters increase, but their wall thickness decreases. Under an individual pressure increase of the outer gas-assisted layer, surface bump defects are generated. Under an individual pressure increase of the inner gas-assisted layer, the diameters of PP micro-catheters swell prominently until they break. Therefore, although DL-GAE can eliminate extrusion problems of polymer micro-catheters, it is suggested that reasonable process parameters for the melt and double assisted gas should be satisfied and matched. This work can provide significant technical support for the DL-GAE of polymer micro-catheters during manufacture.

## 1. Introduction

Polymer micro-catheters have already been widely applied in many fields such as fiber optic communication, automobile oil circuits, and precision instruments, especially those used in interventional medical surgery. In general, polymer micro-catheters are produced using the extrusion molding technique [1]. In the past, research about the extrusion molding of polymer micro-catheters mainly focused on optimal die design [2,3], experiments [4], numerical simulations [5], etc. Due to high viscoelasticity and difficulties caused by the high stress to the die channel of the molten polymer, some effects, including macromolecular chain orientation [6], elastic energy storage recovery [7], and rearrangement of flow velocities [8], are induced, which finally result in the generation of extrusion problems, including extrudate swelling [9], extrusion deformation [10], and melt fracture [11] of the extruded molten polymer, especially during high-speed extrusion. In order to solve these aforementioned problems, some improved methods have been widely investigated by scholars, e.g., polymer modification [12], die fluoride coating [13], ultrasonic-assisted vibration extrusion [14], and gas-assisted extrusion (GAE). GAE is an environmentally friendly, convenient, and high-efficiency process for polymers that has been studied by scholars worldwide. Brzoskowski [15] proposed the GAE technique and applied it to the extrusion molding of rubber compounds. Subsequently, Liang [16] successfully employed GAE in the extrusion molding of polymer sheets. Huang [17,18] experimentally and numerically studied the GAE of round rod plastics. Xiao [19,20] carried out GAE of square-type polymers via experimental and numerical methods. Song and Liu [21,22] performed GAE of polymer profiles with a T-typed cross-section. Jiang [23] numerically and experimentally achieved the GAE molding of sheet plastics. Huang [24] and Deng [25,26] successfully achieved gas-assisted co-extrusion molding of two polymers. Ren [27] successfully achieved compressible GAE molding of polypropylene (PP) using the numerical simulation method.

In the previously reported research, GAE has mainly been focused on solid polymer products. The GAE of hollow, linear-type polymer products has been studied by several scholars. For example, Ren [28] numerically studied the GAE and die design of square-type polymer pipes. Xu [29] performed numerical research on polymer tubes. Yu [30,31] investigated the GAE of polymer water pipes via the numerical method. However, for polymer micro-catheters, few studies have been reported by scholars. Huang [32] performed research on GAE of polymer micro-tubes. Ren [33] successfully achieved GAE of polymer micro-catheters via finite element numerical simulation and was the first to experimentally verify that the extrusion problems of polymer micro-catheters can be effectively eliminated via the GAE method. The GAE of polymer micro-catheters was subsequently studied by a few scholars. For example, Luo [34] improved the inner gas inflation in the GAE of polymer microtubes via the optimized design of the die. Liu [35] improved the structure of gas chambers in the GAE of polymer micro-catheters via experimental and numerical methods. Chen [36] studied the influence of gas inlet slit width on the GAE of polymer micro-tubes. Zhang [37] achieved three-dimensional numerical simulations of the effect of melt viscoelastic rheology on deformation behavior for the GAE of polymer catheters.

**Figure 1:**
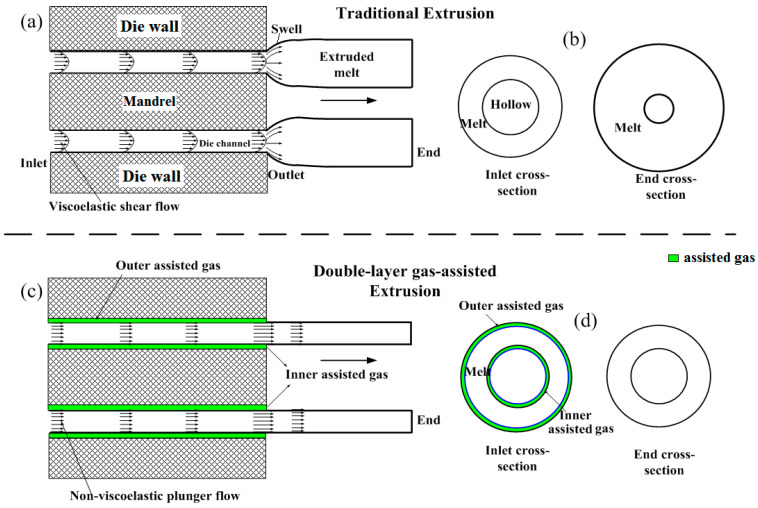
Schematic diagram comparing both extrusions of polymer micro-catheters; (**a**,**c**): axial cross-section of traditional extrusion and DL-GAE, respectively; (**b**,**d**): inlet cross-section and end cross-section of polymer micro-catheters manufactured via traditional extrusion and DL-GAE, respectively.

From the perspective of structure, there is a great difference between hollow polymers and solid polymers. Solid polymers have only one wall, so only one layer of assisted gas is established between the melt and the inner wall of the die channel for the GAE of solid polymers. However, hollow polymers have two walls. Extrusion problems occur at both the outer and inner walls, which results in more serious extrusion problems (see Figure 1a). To solve these extrusion problems, double layers of assisted gas must be injected onto the inner wall and outer wall for the GAE molding of polymer micro-catheters. That is, the GAE of polymer micro-catheters is double-layer gas-assisted extrusion (DL-GAE) (see Figure 1c). By means of two layers of assisted gas, the shear stresses, tensile stresses, and normal stresses of molten polymer designed with a die channel are greatly eliminated, and the flow behavior of molten polymer is transformed from a viscoelastic shear flow (see Figure 1a) to a non-viscoelastic plunger flow (see Figure 1c). As a result, elastic energy storage recovery, the molecular chain orientation effect of molten polymer, and stress concentration on the die outlet can be effectively overcome. Finally, extrusion problems, including extrudate swelling (see Figure 1b), extrusion deformation, and melt fracture in the traditional extrusion of polymer micro-catheters, are effectively resolved by using DL-GAE technology (see Figure 1d).

Although it has already been verified that the GAE method can solve the extrusion problems of extrudate swelling, extrusion deformation, and melt fracture [38], abiding by reasonable process parameters is a prerequisite for this. Moreover, the process parameters and phase models for DL-GAE molding of polymer micro-catheters are more complicated than those for single-layer GAE of solid polymers. If the process parameters are changed, DL-GAE molding of polymer micro-catheters may be impacted [39,40]. However, few studies have reported on the influences of the interaction between the melt and gas on DL-GAE molding of polymer micro-catheters or on reasons for the DL-GAE’s failure. Therefore, to ascertain the influence mechanisms and reasons for failure of DL-GAE molding of polymer micro-catheters, the effects of the melt and gas process parameters (melt flow rate and gas pressure) on the DL-GAE molding of polymer micro-catheters were experimentally and numerically investigated in this study. Because numerical simulations of GAE were used in past studies [17,18,19,20,21,22,24,25,26,28,29,30,32,33], the role of GAE was usually simplified into a full-slip boundary condition [33,41]. This simplified method not only ignores the impact of assisted gas but also cannot effectively reflect the interaction relationships between the melt and gas. To overcome this limitation, the establishment of a gas phase in GAE has been considered by some scholars [27,35,36,39,40]. In this study, to examine the influences of interaction between the melt and double assisted gas on DL-GAE molding of polymer micro-catheters, the finite element geometric model of three-phase flow is established according to the actual situations of DL-GAE molding of polymer micro-catheters.

There are three main purposes in this work: (1) to experimentally investigate the influences of the melt and assisted gas process parameters (melt flow rate and gas pressure) on the DL-GAE molding of polymer micro-catheters; (2) to experimentally and numerically study the interaction impacts between the melt and two layers of gas; and (3) to ascertain the influence mechanisms for the effects of interactions between the melt and gas on the DL-GAE molding of polymer micro-catheters via the finite element numerical method and to analyze the reasons for failure.

In this work, the experimental setup of DL-GAE molding of PP micro-catheters is constructed, and a DL-GAE die is also designed. The influences of melt flow rate and double assisted gas pressure on the DL-GAE molding of PP micro-catheters are experimentally investigated. The experimental results show that with an increase in the melt flow rate, the diameters and wall thickness of PP micro-catheters increase. However, the higher flow rate of the melt impacts the stability of DL-GAE molding of PP micro-catheters. When the melt flow rate increases to a certain high value, DL-GAE failure occurs. As the increase in double assisted gas pressures is synchronized, the diameters of the polymer micro-catheters increase, but their walls become progressively thinner. When an individual increase in the outer assisted gas occurs, surface bumps and corrugation defects in PP micro-catheters are gradually generated. When an individual increase of the inner assisted gas occurs, the diameters of PP micro-catheters increase rapidly until the inner cavity bursts. To ascertain these aforementioned reasons for the failure of DL-GAE molding of PP micro-catheters, the influences of the melt flow rate and double assisted gas pressure on the DL-GAE molding of PP micro-catheters are also numerically studied in this work. The numerical physical fields’ distributions (pressure, flow velocity, shear rate, first normal stress difference) of the melt and double assisted gas are obtained. The numerical results show that the main reason for failure in DL-GAE molding of polymer micro-catheters is that under unreasonable process parameters of the melt and double assisted gas, the flow behaviors of the melt or double assisted gas are unstable due to the greater interaction stresses or forces between the melt and double assisted gas. Abiding by reasonable process parameters and carrying out correct matching between the melt and double assisted gas are crucial for achieving successful DL-GAE molding of polymer micro-catheters.

## 2. Materials and Methods

### 2.1. Setup

**Figure 2:**
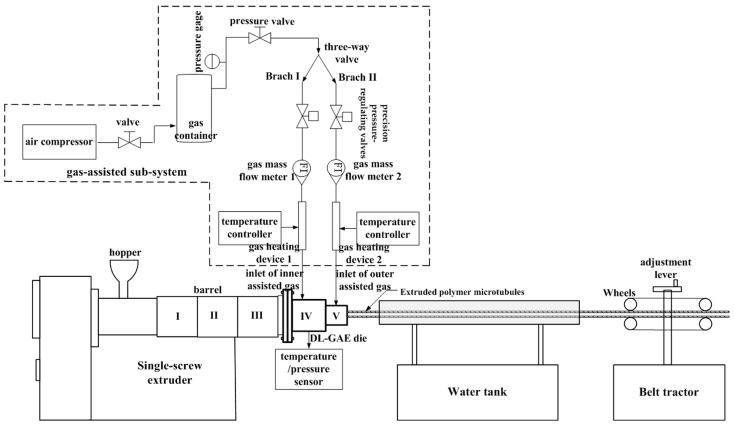
Schematic diagram of experimental system for DL-GAE molding of polymer micro-catheters. In Figure 2, There are three sections of heaters fixed on the surface of extruder barrel, i.e., I, II, and III. There are two sections of heaters fixed on the surface of DL-GAE die, i.e., IV, and V.

To study the influences of the process parameters of the melt and double assisted gas on DL-GAE molding of polymer micro-catheters, a custom-built DL-GAE molding system for polymer micro-catheters was constructed, as presented in Figure 2.

As shown in Figure 2, the experimental system built for DL-GAE molding of polymer micro-catheters consisted of a single-screw extruder, water-cooling tank, belt tractor, GAE die, and gas-assisted subsystem. The screw diameter of the extruder (GRQ25, Huaxi Plastic Machinery Co., Ltd., Dongguan, China) was 25 mm, its length-to-diameter ratio was 25, and its maximum screw speed was about 1450 r/min. A water-cooling tank with two meters was used to cool the extruded melt of the micro-catheters to a natural temperature of about 20 °C via a cycling system of cooling water. A belt tractor was employed to drag the extruded micro-catheters. The traction velocity was adjustable via the variable frequency motor, and its maximum traction velocity was about 30 m/min.

In the experiments, the DL-GAE technique was employed to eliminate the extrusion problems of polymer micro-catheters. To achieve this aim, a gas-assisted subsystem was built, shown in Figure 2. The gas-assisted subsystem included an oil-free air compressor (LD-80L, 800 W × 3, Juba Co., Taizhou, China), valves, a gas container with a volume of 0.4 m^3^ (1V-3/8, Nanchang, China), a pressure-reducing valve (AFC2000, AirTAC Co., Taiwan), a three-way valve, two precision pressure-regulating valves (SMC IR1000-01BG, Wuhan, China), two gas flow meters (LZB-6/LZB-40, Yuyao, China), two gas heating devices and a temperature controller (2 kW, Lai Heng Co., Ltd., Shanghai, China), PTFE pipelines, and gas pipe joints. The gas container was used to load the compressed air produced by the air compressor and to ensure the stability of the assisted gas flow during the DL-GAE of polymer micro-catheters. Two gas heating devices were used to heat the double layers of assisted gas. The temperature of the double assisted gas was configured and controlled by the temperature controller.

**Figure 3:**
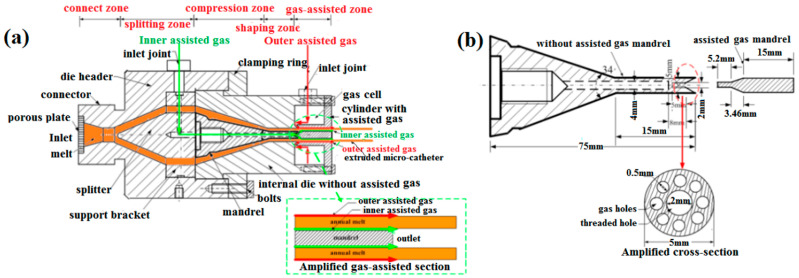
Structure diagram of DL-GAE die for polymer micro-catheters: (**a**) axial sectional view, (**b**) structure of inner gas assistance.

GAE die is one of the most important and key components in the DL-GAE of polymer micro-catheters. In this study, a novel DL-GAE die was optimally designed. Compared with the GAE die reported previously [33], the structure of the DL-GAE die was modified and more convenient to assemble. A structure diagram of the novel DL-GAE die is shown in Figure 3a. As can be observed in Figure 3a, the DL-GAE die includes five zones: the connecting zone, splitting zone, compression zone, shaping zone, and gas-assisted zone. The DL-GAE die was connected with the barrel outlet of the extruder via the connector with male thread and via the connection flange with female thread. The hollow cavity of the melt was formed by a splitter, support bracket, and mandrel. The splitter and the mandrel were fixed on the support bracket via the thread connection method. The internal die without gas assistance was sleeved around the mandrel and fixed on the die header. The cylinder with gas assistance was fixed on the internal die without gas assistance via bolts. Moreover, an annular gas cell was formed by the cylinder with gas assistance and the internal die without gas assistance; this cell was utilized to buffer the direct shock of the outer assisted gas. In addition, there was a slit of about 0.2 mm width between the internal die without gas assistance and the cylinder with gas assistance. It is worth mentioning that the angle of the slit was designed to be about 30°. This type of modified design can not only reduce the stress impact of outer assisted gas on the annular melt but can also prevent molten polymer from flowing into the gas cell when the DL-GAE die is horizontally placed during the DL-GAE molding of polymer micro-catheters.

In DL-GAE die, in order to form the inner gas-assisted layer between the outer surface of the mandrel and the inner surface of the melt, a novel structure was first designed. A structure diagram of the novel design of a mandrel for inner gas assistance is shown in Figure 3b, in which one can observe that the mandrel was divided into two sections: non-gas-assisted and gas-assisted. These two sections could be connected to the whole mandrel by using the male and female threads. To facilitate the formation of the inner gas-assisted layer, there was also a slit with a width of about 0.2 mm and an angle of 30° between the two sections of mandrel. To intake the inner assisted gas flow into the gas-assisted zone and fasten the gas-assisted mandrel, the non-gas-assisted mandrel was specially designed; its cross-section is shown in Figure 3b, which shows how the eight gas channels and the threaded hole with female thread were manufactured. In the DL-GAE die, the basic paths of the inner gas assistance and outer gas assistance are labeled with green lines and red lines in Figure 3a, respectively. Photos of the assembled DL-GAE die and its components are provided in Figure S1 of the Supplementary Materials section.

To obtain the molten PP, the temperatures of three sections of the barrel and two sections of the DL-GAE die were configured according to the values provided in Table 1. At the same time, in the experiments of DL-GAE molding of PP micro-catheters, when the temperatures of the barrel and DL-GAE die reached the preset temperatures, the inner assisted gas and the outer assisted gas were injected into the die channel in advance, before starting the rotation of the extruder screw.

### 2.2. Numerical Analysis

#### 2.2.1. Models

**Figure 4:**
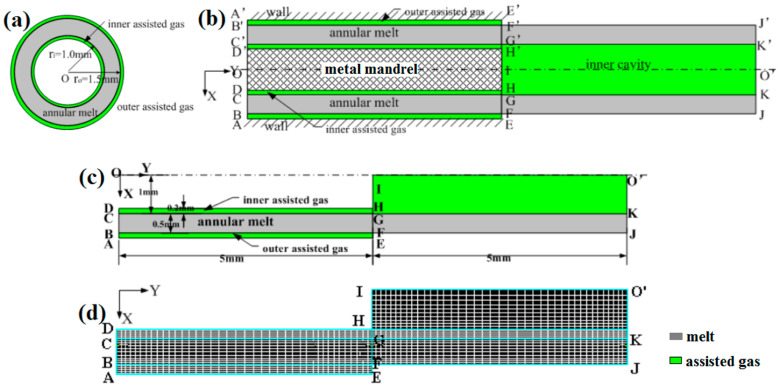
Geometric models of DL-GAE of micro-catheters: (**a**) cross-section at inlet face; (**b**) schematic diagram of DL-GAE; (**c**) half of axis-symmetric model; (**d**) quadrilateral mesh. In Figure 4, the letters of A-H, and A’-H’ are the boundary points of model, which are utilized to define the boundary condition in Section 2.2.3.

To reasonably simplify the numerical computing, the melt shaping section in the die and the extruded melt section were considered in finite element numerical simulations. The geometric models of DL-GAE molding for polymer micro-catheters are presented in Figure 4. Figure 4a provides the cross-section of the inlet face, the inner radius, the outer radius, and the wall thickness of the polymer micro-catheters; these were 1.0 mm, 1.5 mm, and 0.5 mm, respectively. Figure 4b shows a two-dimensional geometric model with an axis-symmetric structure for the DL-GAE molding of polymer micro-catheters. For this work, the geometric model of a sandwich-like, three-phase flow was built. Double gas-assisted layers (with widths of 0.2 mm) were constructed; the model was 5 mm long and had die inside and outside. Moreover, the inner cavity of polymer micro-catheters connected with an inner gas-assisted layer was also considered to be in the zone of the outside die. According to the axis-symmetric property, half of the geometric model was utilized to increase the speed of the numerical simulation, which is presented in Figure 4c. Figure 4d shows the quadrilateral mesh used in Figure 4c. The mesh was divided into 1200 finite elements.

#### 2.2.2. Numerical Equations

In the simulations, the numerical computing for DL-GAE of polymer micro-catheters was performed via Ansys Polyflow^®^. Several hypotheses should be applied in the simulations: (1) The molten polymer is non-Newtonian fluid with a steady-state, non-isothermal, and laminar flow. The two layers of assisted gas are defined as Newtonian fluid with a steady state and a non-isothermal layer; (2) Gravity and the inertia force of the molten polymer are neglected because the polymer has a higher viscoelasticity and a slow velocity. The gravity of the double assisted gas is also neglected due to its light weight, but its inertia forces are considered due to the high flow velocities; (3) The osmosis of assisted gas into the molten polymer is not considered.

The continuity, momentum, and energy equations [39] are presented in Equations (1)–(3), respectively:

(1) $$\nabla \cdot \rho_{k}v_{k}=0$$

(2) $$\rho_{k}v_{k}\cdot \nabla v_{k}+\nabla \rho_{k}-\nabla \cdot \tau_{k}=0$$

(3) $$\rho_{k}C_{\mathrm{pk}}v_{k}\cdot \nabla T_{k}+k_{k}\nabla^{2}T_{k}-\tau_{k}:\nabla v_{k}=0$$

Here, ∇ denotes the Hamilton operator, $\rho_{k}$ is density, $v_{k}$ is velocity, $p_{k}$ is pressure, and $\tau_{k}$ is the extra stress tension. $C_{\mathrm{pk}}$ is the specific heat capacity and $T_{k}$ is temperature. $k_{k}$ is heat conductivity. $\tau_{k}:\nabla v_{k}$ is the term of viscous dissipation. The subscript *k* represents the melt (*I*) or double assisted gas (*II*).

As for the constitutive model of molten polymer, the Phan–Thien–Tanner (PTT) equation [33,35,36] is employed:

(4) $$\tau_{I}=\tau_{I1}+\tau_{I2}$$

(5) $$\begin{array}{l} \exp\left[\frac{\varepsilon \lambda }{(1-\eta_{Ir})\eta_{I1}}tr(\tau_{I1})\right]\tau_{I1}+\lambda \left[\left(1-\frac{\xi }{2}\right){\overset{\nabla }{\tau_{I}}}_{1}+\frac{\xi }{2}{\overset{\Delta }{\tau }}_{I1}\right]\\ =2(1-\eta_{I1})\eta_{I1}D_{I} \end{array}$$

(6) $$\tau_{I2}=2\eta_{I2}D_{I}$$

where *ε* and *ξ* are related to the tensile and shear characteristics of the melt, respectively. ${\overset{\nabla }{\tau_{I}}}_{1}$ and ${\overset{\Delta }{\tau_{I}}}_{1}$ are the upper-convected and lower-convected time derivatives of extra stress tension, respectively. λ is the melt’s relaxation time. *η_Ir_* is the melt’s viscosity ratio, i.e., *η_I_*_2_/*η_I_*_1_. *η_I_* is the melt’s total viscosity. *η_I_*_1_ and *η_I_*_2_ are the melt’s non-Newtonian viscosity and Newtonian viscosity, respectively. $D_{I}$ is the melt’s tensor deformation rate.

As for two layers of assisted gas, their constitutive equations are written in Equations (7) and (8):

(7) $$D_{\mathrm{II}}=\frac{1}{2}\left(\nabla v_{\mathrm{II}}+\nabla^{T}v_{\mathrm{II}}-\frac{1}{3}\nabla v_{\mathrm{II}}\delta_{\mathrm{II}}\right)$$

(8) $$\tau_{\mathrm{II}}=2\eta_{\mathrm{II}}D_{\mathrm{II}}$$

where $\delta_{\mathrm{II}}$ is the second-order unit tensor. $\eta_{\mathrm{II}}$, $\tau_{\mathrm{II}}$, and $D_{\mathrm{II}}$ are the double assisted gas’s viscosity, inelastic stress tensor, and tensor deformation rate, respectively.

Due to the consideration of non-isothermal characteristic, the Arrhenius equation [42] is utilized to express the relationship between the melt’s viscosity and temperature:

(9) $$\eta_{I}^{\prime }=\eta_{I}\cdot H\left(T_{I}\right)$$

(10) $$H\left(T_{I}\right)=\exp\left[\frac{E_{{\dot{\gamma }}_{I}}}{R}\left(\frac{1}{T_{I}-T_{0}}-\frac{1}{T_{r}-T_{0}}\right)\right]$$

where $H\left(T_{I}\right)$ is the Arrhenius function. $T_{I}$ is the temperature. $T_{r}$ is the reference temperature, which is equal to the temperature of the melt, i.e., 473 K. $E_{{\dot{\gamma }}_{I}}$ is the viscous activation energy. R is the gas constant, i.e., *R* = 8.314. For double assisted gas, the influence of temperature on viscosity is neglected because the viscosity of this gas is extremely low, and the viscosity changes are few for the Newtonian fluid.

#### 2.2.3. Boundary Conditions

Based on Figure 4c, the boundary conditions were set as follows:

(1) Inlets: BC, AB, and CD are the inlets of the melt, inner assisted gas, and outer assisted gas, respectively. The fully developed flow was considered for the melt and double assisted gas flow in the shaping section of the die; these inlets obey kinetic equations, i.e., *v_x_* = 0 and ∂*v_y_*/∂*y* = 0 where *v_x_* and *v_y_* are the radial and axial velocities, respectively. The constant temperatures were set on all inlets.
(2) Walls: AE, DH, and HI are the walls of the die and mandrel, respectively. The no-slip condition was set on the walls. In addition, the temperature of 473 K was set on all walls.
(3) Free boundary: FJ is the free boundary of the extruded micro-catheters’ outside die. There are no forces on the free boundary. In addition, the heat flux boundary condition between the extruded micro-catheter and the air environment was considered. The heat flux equation [43], i.e., d*Q_I_* = *α_I_*_1_ (*T_I_* − *T_α_*_1_), was employed here, where *T_I_* and *T_α_*_1_ are the temperatures of the melt and air environment, respectively, and *T_α_*_1_ = 300 K. *α_I_*_1_ = 5 is the heat transfer coefficient.
(4) Interfaces: BF and CK are the interfaces between the melt and the double assisted gas. If one assumes that there is no relative slippage on the interfaces, the dynamic and kinetic equations are obeyed, i.e., *f_In_* = *f_IIn_*, *f_Is_* = *f_IIs_*, *v_Is_* = *v_IIs_*, and $\vec{v}\cdot \vec{n}=0$ where *f_n_* and *f_s_* are the normal and tangential forces, respectively. $\vec{n}$ is the normal unit vector. On the interfaces, the heat flux is also considered, i.e., d*Q_I_* = d*Q_II_* = α*_I_*_2_ (*T_I_* − *T_α_*_2_). Here, α*_I_*_2_ is set to 10 due to strong heat exchange. *T_α_*_2_ is set to 473 K.
(5) Symmetry axis: IO’ is the symmetry axis.
(6) Ends: KJ, EF, and KO’ are the ends of the melt, outer assisted gas, and inner assisted gas, respectively. If the traction force of the melt is considered in the simulation, a certain normal force or velocity can be imposed on the end of the melt. The outflow thermal boundary condition was imposed on the ends of the melt and gas because the temperatures could not be known in advance.

#### 2.2.4. Material Parameters

Polypropylene (PP) (H-T03-L, SINOPEC Co., Maoming, China) was utilized in the experiments. The density of the PP was approximately 920 kg/m^3^, and its melt index was approximately 3 g/10 min (ISO1133-1). Moreover, in the experiments, except for PP, which was molten due to the heating, no other polymer materials or additives were added into the molten PP. Therefore, there were no reactions occurring during the experiments. Table 2 lists the numerical material and rheological configurations of molten PP and double assisted gas [33,36].

## 3. Results and Discussion

### 3.1. Influence of Melt Flow Rate

**Figure 5:**
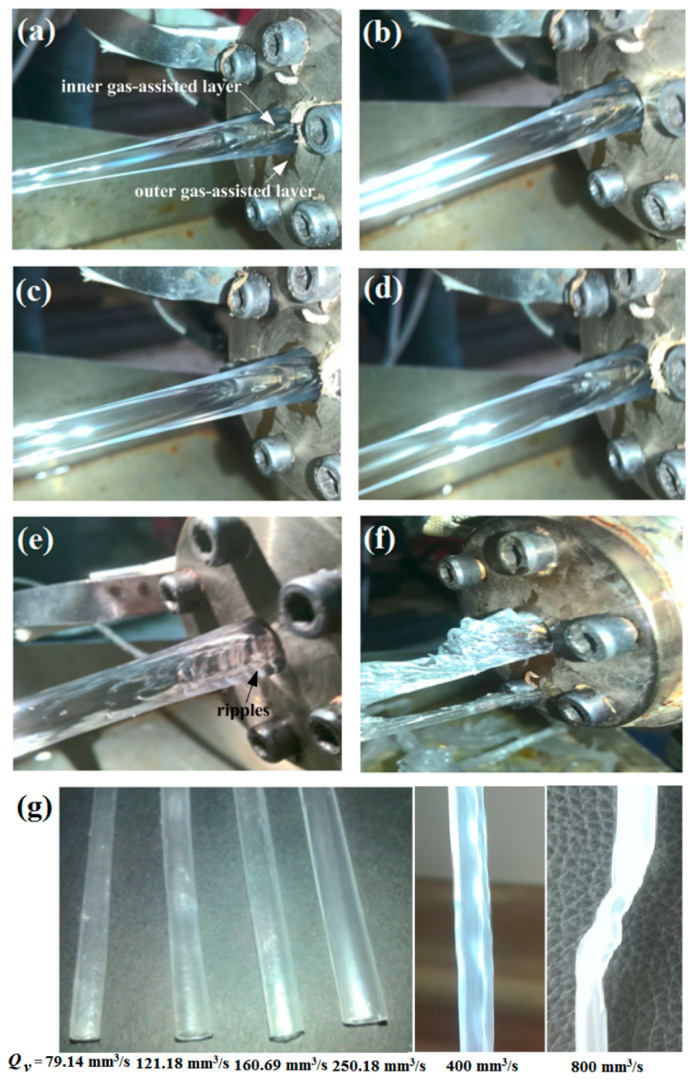
PP micro-catheters extruded via DL-GAE under various melt flow rates. (**a**) *Q_v_* = 79.14 mm^3^/s; (**b**) *Q_v_* = 121.18 mm^3^/s; (**c**) *Q_v_* = 160.69 mm^3^/s; (**d**) *Q_v_* = 250.18 mm^3^/s; (**e**) *Q_v_* = 400 mm^3^/s; (**f**) *Q_v_* = 800 mm^3^/s; (**g**) the extruded PP micro-catheters under different melt flow rates.

To investigate the influence of the melt flow rate (*Q_v_*) on DL-GAE molding of PP micro-catheters, stable DL-GAE was achieved in advance using the following reasonable process parameters: the screw speed was about 4.86 r/min; the temperatures of the melt and gas were 220 °C; the inner assisted gas pressure and outer assisted gas pressure were 0.08 MPa and 0.15 MPa, respectively; and the traction speed of the micro-catheters was 25 mm/s. Figure 5a–f presents the PP micro-catheters extruded via DL-GAE at various melt flow rates. The extruded PP micro-catheter products under different melt flow rates are presented in Figure 5g.

As shown in Figure 5a–d, under reasonable process parameters for the melt and gas, stable DL-GAE molding of PP micro-catheters can be achieved. At this time, the double assisted gas is stably formed. Moreover, the size of the PP micro-catheters gradually increases with an increase in the melt flow rate. However, when the melt flow rate increases to about 400 mm^3^/s, the stability of the double gas-assisted layers is impacted. At this time, it is difficult for the inner and outer gas-assisted layers to form, and the ripple phenomenon occurs on the surface of the micro-catheters, at the die outlet, due to the influence of assisted gas (see Figure 5e). As a result, when the melt continuously increases, the double gas-assisted layers cannot be formed at all. In this case, the DL-GAE of PP micro-catheters failed completely (see Figure 5f); even if the melt flow rate was reduced, the stability of the DL-GAE of the PP micro-catheters cannot be recovered again.

**Figure 6:**
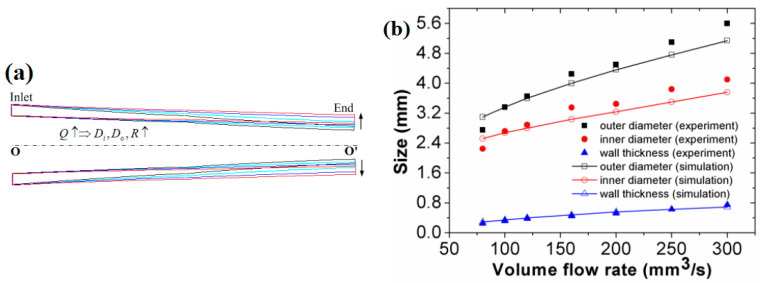
Numerically simulated polymer micro-catheters at different melt flow rates: (**a**) profile change; (**b**) size change comparison between the numerical simulation and experiment. In Figure 6a, the sizes of micro-catheters increase with the melt flow rate, the profiles marked with black, green, blue, and red color, respectively.

Based on the process parameters in the experiments, as well as on the traction speed of 30 mm/s at the end of melt, the DL-GAE molding of polymer micro-catheters under different flow rates was numerically simulated. The change in the extruded profiles of the polymer micro-catheters manufactured via DL-GAE at different melt flow rates is presented in Figure 6a.

In Figure 6a, one can observe how under fixed process parameters for the melt and double assisted gas, when the flow rate of the melt increases, the outer diameter, inner diameter, and wall thickness of the polymer micro-catheters gradually increase. Compared with the sizes of extruded PP micro-catheters at different melt flow rates provided in Figure 5g, the numerical results of the profile change for micro-catheters are in good agreement with the results of the experiments. To further verify the results, the size changes (outer diameter, inner diameter, and wall thickness) of polymer micro-catheters, measured via finite element numerical simulation and experiments, were all obtained and compared, as presented in Figure 6b. From Figure 6b, it can be observed that under the same process parameters and boundary conditions, the sizes of polymer micro-catheters extruded via DL-GAE increase with increasing melt flow rates. Moreover, the size change trends of polymer micro-catheters based on DL-GAE for numerical simulation are the same as those in the experiments. The size change trends of polymer micro-catheters are related to the increase in the melt flow rate in unit time.

**Figure 7:**
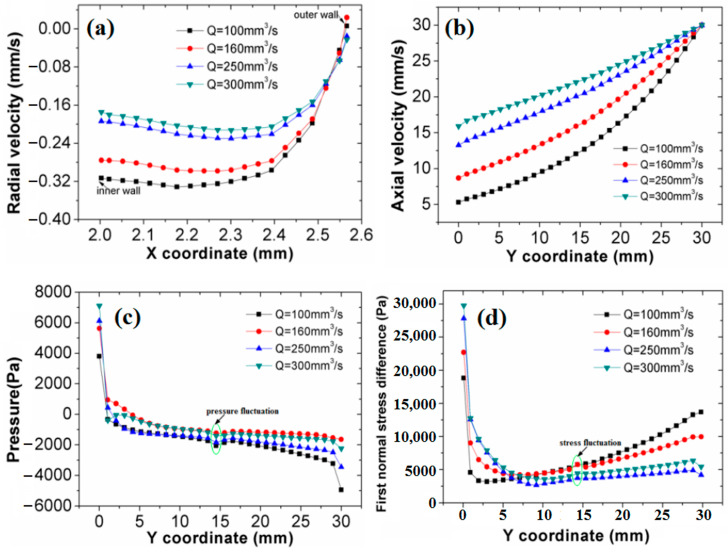
Physical field distributions of melt at various flow rates: (**a**) radial velocity; (**b**) axial velocity; (**c**) pressure; (**d**) first normal stress difference.

To further explore the influence of the melt flow rate on the DL-GAE of polymer micro-catheters, the flow velocities, pressure, and first normal stress difference (N_1_) distributions of the melt at various flow rates were obtained; these are presented in Figure 7a–d.

Figure 7a is the radial flow velocity distribution of the melt in the radial direction of the die outlet. In Figure 7a, the radial flow velocity distribution of the melt is non-uniform, i.e., the melt’s negative radial velocity near the inner wall is greater than that of the melt near the outer wall. This occurs because the molten micro-catheter was pulled by a traction speed, which induces the melt flow towards the radial direction of the inner wall and, finally, shrinks the sizes of the polymer micro-catheters. As the melt flow rate increases, the sizes of the micro-catheters increase due to the decreased negative radial velocity of the melt. Figure 7b shows the axial flow velocity distribution of the melt. In Figure 7b, the melt’s axial flow velocity gradually increases under the role of axial traction. Moreover, the melt’s axial velocity increases with the flow rate, which expands the size of the polymer micro-catheters. Figure 7c presents the melt’s axial pressure distribution at various flow rates. The melt’s pressure gradually reduces along the axial direction, especially at the inlet of the melt, where the pressure drop is sharp. Moreover, a sudden drop in pressure is generated at the die outlet; the pressure drop produces unstable extrusion. With an increase in the melt flow rate, the pressure of the melt increases for the DL-GAE of polymer micro-catheters. Figure 7d provides the melt’s first normal stress difference (N_1_) distributions. In Figure 7d, N_1_ mainly focuses on the die inlet due to the increased shear stress and normal stress generated by double assisted gas, and it rapidly decreases near the inlet of the die channel and then slightly increases along the axial direction due to the axial dragging effect. Moreover, N_1_ increases with the melt flow rate at the channel inlet, but, for the melt’s outside die, N_1_ weakens slightly due to the radial expansion effect of polymer micro-catheters.

**Figure 8:**
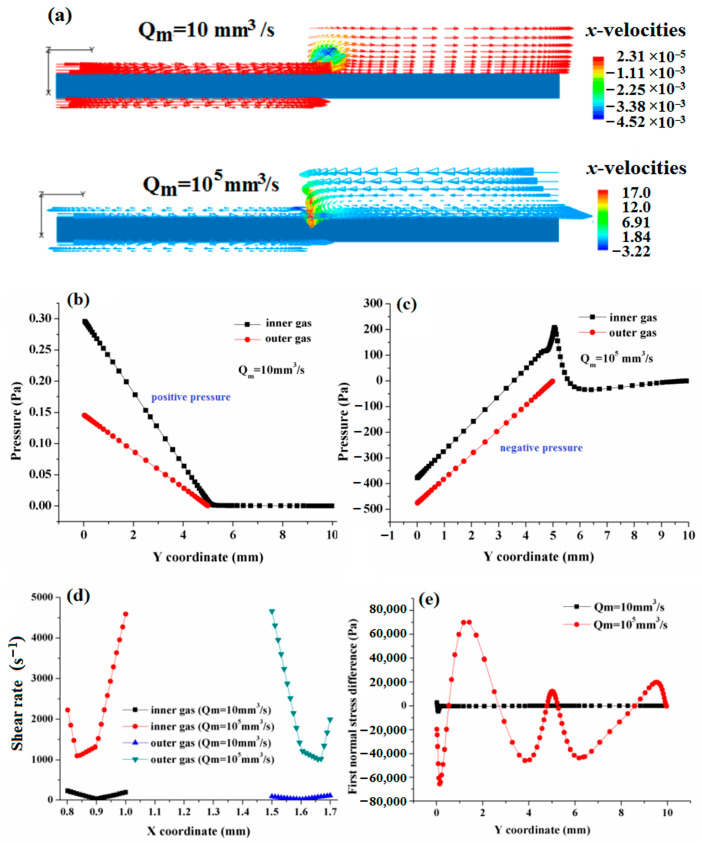
Comparison of the influence of melt flow rate on DL-GAE molding of polymer micro-catheters: (**a**) velocity field of assisted gas; (**b**) pressure axial distribution curves of double assisted gas at regular melt flow rate; (**c**) pressure axial distribution curves of double assisted gas at higher melt flow rate; (**d**) shear rate radial distribution; (**e**) first normal stress axial distribution curves.

With increasing melt flow rate, the greater shear stress and N_1_ imposed by the melt impacts the stability of the flow behavior of double assisted gas. In the experiments, it was found that under fixed pressures of double assisted gas, with an increasing melt flow rate, the double assisted gas channels are gradually filled with melt. When the melt flow rate increases to a certain greater value, the melt fills up the entire die channel, finally inducing the failure of the DL-GAE. To ascertain the effect of the higher melt flow rate on the failure of DL-GAE molding of polymer micro-catheters, the flow velocity, pressure, shear rate, and stress distributions of the double assisted gas were obtained and compared in terms of the regular melt flow rate and higher flow rate; these are presented in Figure 8a–e.

Although the experimental phenomenon of gas channels becoming filled with melt was not numerically simulated here, some reasonable interpretations can be obtained from the numerical results presented in Figure 8. Reverse flow behavior (see Figure 8a) and negative pressure (see Figure 8c) of the double assisted gas occurred in numerical simulations of greater flow rates of the melt, which can indicate that stable flow behavior in the double assisted gas is not formed when the flow rate of the melt is higher; that is, the DL-GAE of the polymer micro-catheter fails at this time. The main reason for this is that the shear rates (see Figure 8d) between the melt and double assisted gas are high for the higher melt flow rate, which makes the first normal stress (see Figure 8e) increase and fluctuate along the gas channel. Finally, the stability of the double assisted gas is destroyed.

### 3.2. Influence of Gas Pressure

#### 3.2.1. Synchronized Influence of Double Assisted Gas Pressures

**Figure 9:**
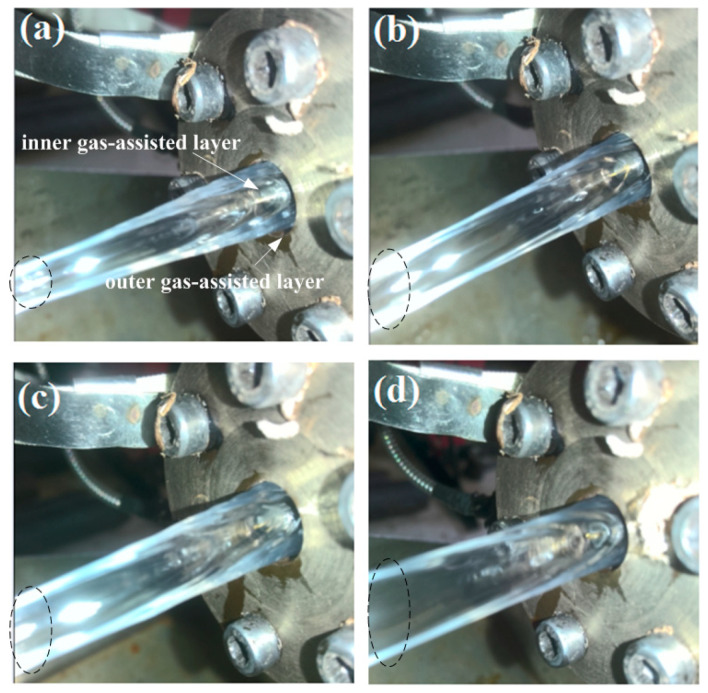
Synchronized influence of double assisted gas pressures on DL-GAE molding of PP micro-catheters via experiments. (**a**) *P_i_* = 0.06 MPa, *P_o_* = 0.12 MPa; (**b**) *P_i_* = 0.065 MPa, *P_o_* = 0.13 MPa; (**c**) *P_i_* = 0.07 MPa, *P_o_* = 0.14 MPa; (**d**) *P_i_* = 0.075 MPa, *P_o_* = 0.15 MPa. The sizes change of PP micro-catheters at different double assisted gas pressures are marked in Figure 9a–d with black circles.

In the DL-GAE molding of PP micro-catheters, the stable establishment of double gas-assisted layers (i.e., inner gas-assisted layer and outer gas-assisted layer) is one of the most important and key factors. In addition, reasonable pressures of the double assisted gas are vital. In our experiments, the pressures of the double assisted gas could be adjusted via precision pressure-regulating valves. The impact of double assisted gas pressures on DL-GAE molding of PP micro-catheters was experimentally investigated. Firstly, the synchronized influence of double assisted gas pressures was studied. After a stable DL-GAE was formed, the pressures of the double assisted gas (*P_i_* and *P_o_*) were synchronously increased. The PP micro-catheters extruded via DL-GAE under various pressures of double assisted gas are shown in Figure 9a–d.

As shown in Figure 9, with a synchronous increase in double assisted gas pressures, the diameters of PP micro-catheters gradually increased, but their walls gradually became thinner. When the pressure of the inner assisted gas was increased to approximately 0.1 MPa, the PP micro-catheters were blown out because the thinned wall could not withstand the inner cavity’s pressure.

**Figure 10:**
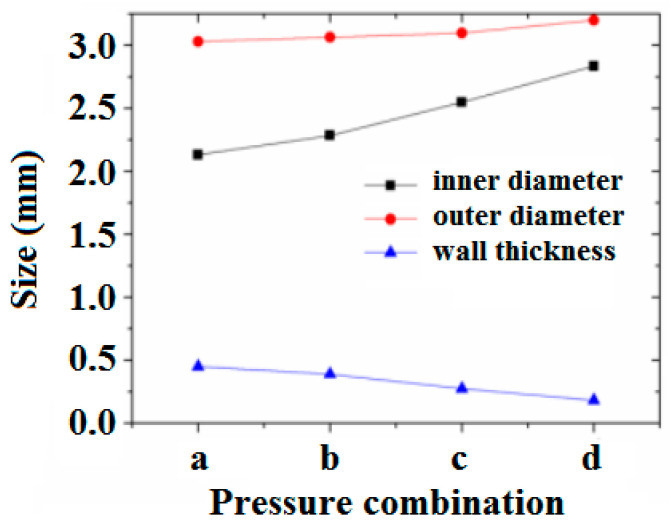
The size changes (inner diameter, outer diameter, and wall thickness) of polymer micro-catheters with synchronized pressure increases of double assisted gas via numerical simulation. In the axial coordination, a–d correspond to the four synchronized pressure increases of double assisted gas provided in Figure 9a–d.

The synchronized impact of double assisted gas pressures on DL-GAE molding of polymer micro-catheters was numerically simulated. The melt flow rate was fixed at 100 mm^3^/s in the simulation. The synchronized pressure increases were exerted on two inlets of double assisted gas, and the size changes (inner diameter, outer diameter, and wall thickness) of the polymer micro-catheters under different synchronized pressures of double assisted gas are presented in Figure 10. The morphologies of the polymer micro-catheters for the synchronized pressure increase of the double assisted gas were also obtained via finite element numerical simulation; these are shown in Figure S2 of the Supplementary Materials section.

In Figure 10, it can be clearly observed that the inner diameter and outer diameter of micro-catheters both increase, but the wall thickness is reduced by a synchronized increase in double assisted gas pressures.

**Figure 11:**
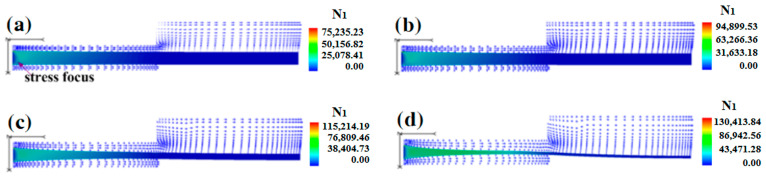
First normal stress differences of melt under various synchronized gas pressures. (**a**) *P_i_* = 0.06 MPa, *P_o_* = 0.12 MPa; (**b**) *P_i_* = 0.065 MPa, *P_o_* = 0.13 MPa; (**c**) *P_i_* = 0.07 MPa, *P_o_* = 0.14 MPa; (**d**) *P_i_* = 0.075 MPa, *P_o_* = 0.15 MPa.

In order to discover the reason for the aforementioned synchronized influence of double assisted gas pressures on the morphology of polymer micro-catheters, the numerical results of melt N_1_ under various synchronized gas pressures were obtained; these are presented in Figure 11a–d.

As shown in Figure 11a–d, at the fixed melt flow rate, the channels of double assisted gas were increasingly wider with a synchronized increase in double assisted gas pressures, which made the melt wall progressively thinner. At the same time, the outside die of the inner cavity space of the polymer micro-catheters also increased, which resulted in an increase in the inner and outer diameters. In addition, with the synchronized increase in double assisted gas pressures, the N_1_ of the melt imposed by the double assisted gas increased at the die inlet, which reduced the wall thickness of the polymer micro-catheters and increased their diameter.

#### 3.2.2. Individual Influence of Outer Assisted Gas Pressure

**Figure 12:**
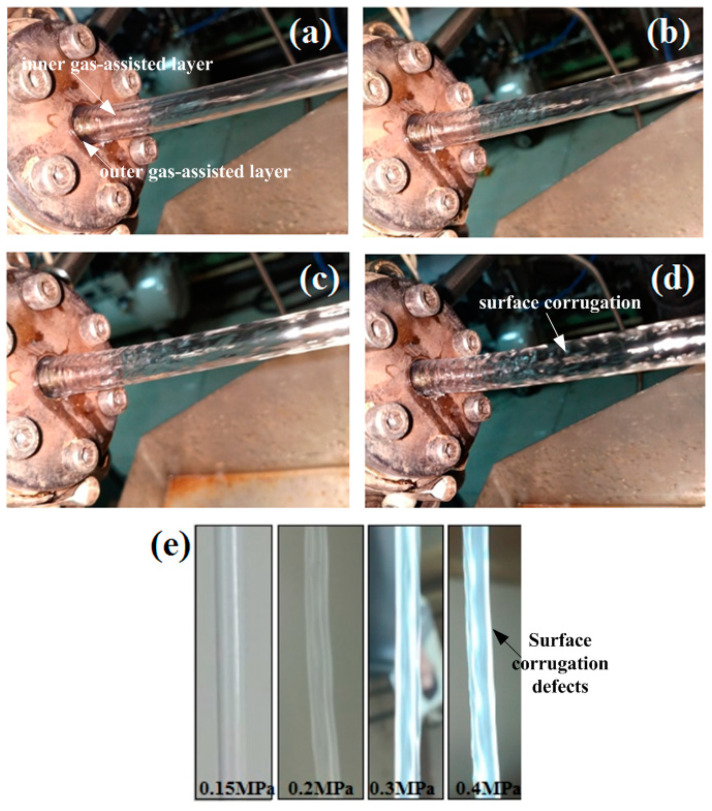
Experimental results for the individual impact of outer assisted gas pressure on DL-GAE of PP micro-catheters. (**a**) *P_o_* = 0.15 MPa; (**b**) *P_o_* = 0.2 MPa; (**c**) *P_o_* = 0.3 MPa; (**d**) *P_o_* = 0.4 MPa; (**e**) prepared PP micro-catheters at various pressures of outer assisted gas.

The individual impact of outer assisted gas pressure on DL-GAE molding of PP micro-catheters was experimentally studied. When the DL-GAE of PP micro-catheters was stable under reasonable process parameters, only the pressure of the outer assisted gas changed; the other process parameters were fixed. Figure 12a–d presents the PP micro-catheters extruded via DL-GAE using various pressures of outer assisted gas.

It can be observed in Figure 12a that the result of DL-GAE molding of micro-catheters was very good when the pressure of the outer assisted gas was approximately 0.15 MPa. With an individual increase in the outer assisted gas pressure, the surface evenness of the PP micro-catheters was gradually destroyed (see Figure 12b–d). The PP micro-catheters extruded via DL-GAE were prepared at various outer assisted gas pressures, which are presented in Figure 12e. In Figure 12e, it can be observed that the quality of the PP microtubule was satisfactory, due to its smooth surface and uniform size, when manufactured under an outer assisted gas pressure of 0.15 MPa. However, as an individual increase in outer assisted gas pressure occurs, the surface bumps and corrugation defects of PP micro-catheters become increasingly severe.

**Figure 13:**
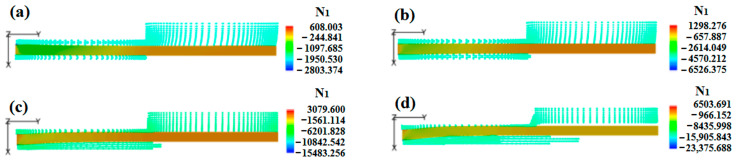
Numerical results for individual influence of outer assisted gas pressure on morphologies and N_1_ change of polymer micro-catheters. (**a**) 0.005 MPa, (**b**) 0.01 MPa, (**c**) 0.02 MPa, (**d**) 0.03 MPa.

The individual effect of outer assisted gas pressure on DL-GAE molding of polymer micro-catheters was also investigated via finite element numerical simulation. In the simulations, the pressure of the outer assisted gas was increased from 0.005 MPa to 0.03 MPa with a fixed pressure of the inner assisted gas. The individual effect of the outer assisted gas pressure on changes in the morphology of the polymer micro-catheters and the numerical results for the melt N_1_ under different outer assisted gas pressures are presented in Figure 13a–d.

In Figure 13a–d, it can be observed that as the outer assisted gas pressure increases, N_1_ of the melt exerted by the outer assisted gas increases at the die entrance, which forces the melt in the die channel to gradually shrink. During a dynamic DL-GAE process, shrinkage of polymer micro-catheters occurs; as a result, axial surface bumps and corrugation defects form under conditions of a higher outer assisted gas pressure. The numerical results, presented in Figure 13a–d, are in good agreement with the experimental results (see Figure 12a–e).

#### 3.2.3. Individual Influence of Inner Assisted Gas Pressure

**Figure 14:**
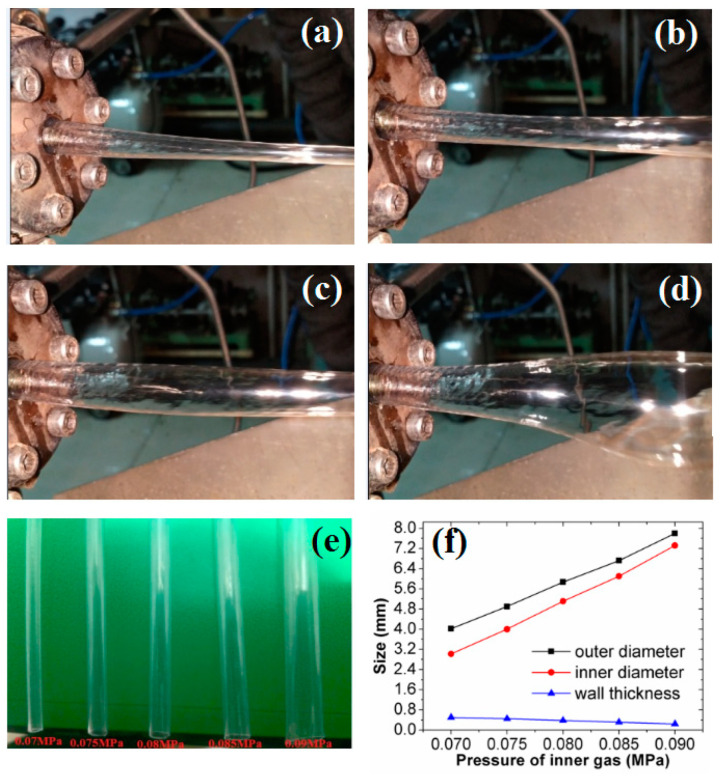
Experimental results for individual impact of inner assisted gas pressure on DL-GAE molding of PP micro-catheters. (**a**) *P_i_* = 0.07 MPa; (**b**) *P_i_* = 0.08 MPa; (**c**) *P_i_* = 0.09 MPa; (**d**) *P_i_* = 0.1 MPa; (**e**) prepared PP micro-catheters; (**f**) sizes change of PP micro-catheters at different pressures of inner assisted gas.

The individual influence of the inner assisted gas pressure on DL-GAE molding of PP micro-catheters was also experimentally investigated. After the DL-GAE of PP micro-catheters was stabilized, the other process parameters were fixed, but the pressure of the inner assisted gas was changed. The PP micro-catheters extruded via DL-GAE under various pressures of inner assisted gas are provided in Figure 14a–d.

It can be observed in Figure 14a that the result of DL-GAE of micro-catheters is very good under an inner assisted gas pressure of approximately 0.07 MPa. With a gradual increase in the pressure of the inner assisted gas, the size of the micro-catheter’s inner cavity gradually swelled (as shown in Figure 14b–d). When the inner assisted gas pressure rose to approximately 1.0 MPa, the PP microtubule was blown out. Moreover, with a continuous rise in the inner assisted gas pressure, the frequency of blow-outs of the inner cavity gradually increased. Figure 14e presents the PP micro-catheters prepared using different pressures of inner assisted gas. Figure 14f provides the size changes of prepared PP micro-catheters, i.e., inner diameter, outer diameter, and wall thickness. In Figure 14e,f, it can be observed that with the individual increase in inner assisted gas pressure, the inner and outer diameters of the PP micro-catheters swell linearly, but the wall thickness reduces linearly due to a greater increase in the degree of the inner diameter.

**Figure 15:**
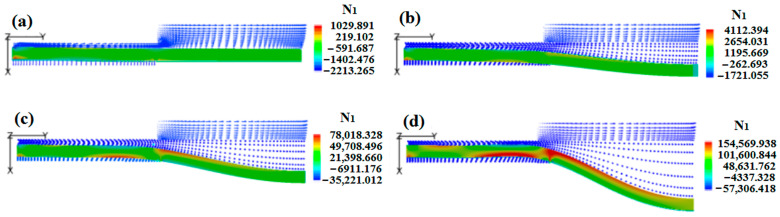
Numerical results of individual impact of inner assisted gas pressure on morphologies and N_1_ change of polymer micro-catheters. (**a**) 0.03 MPa, (**b**) 0.06 MPa, (**c**) 0.1 MPa, (**d**) 0.2 MPa.

The individual impact of inner assisted gas pressure on DL-GAE molding of polymer micro-catheters is also numerically simulated. Under the same pressure of outer assisted gas, the inlet pressure of inner assisted gas increases from 0.003 MPa to 0.02 MPa. The numerical results of the individual impact of inner assisted gas pressure on the morphologies and N1 change of the micro-catheters were obtained; these are shown in Figure 15a–d.

Figure 15 shows how the first normal stress of the melt imposed by the inner assisted gas increases at the outlet of the die and at the inner wall of the micro-catheter’s outside die as the inner assisted gas pressure increases; this forces the molten polymer to flow toward the outer layer. Moreover, as the inner assisted gas pressure gradually increases, the swelling of the inner cavity becomes more prominent. When the inner assisted gas pressure reaches or exceeds the tolerance of the melt wall, the inner cavity of the polymer micro-catheters breaks; these findings are in good agreement with the experimental results provided in Figure 14a–d.

## 4. Conclusions

Although extrusion problems in the manufacture of polymer micro-catheters, including extrudate swelling, deformation, and melt fracture, can be eliminated via DL-GAE technology, some conditions should be satisfied; in particular, the process parameters of the melt and of the double assisted gas should be reasonable. To investigate the phenomenon of instability and the reasons for failures in DL-GAE of polymer micro-catheters, the effects of interaction between the melt and double assisted gas on the DL-GAE molding of polymer micro-catheters were experimentally and numerically studied in this work. The reasons for the failure in DL-GAE of polymer micro-catheters were also numerically analyzed. The numerical results are in good agreement with those of the experiments. The following conclusions were obtained:

(1) As the melt flow rate increases, the diameter and wall thickness of the polymer micro-catheters increase. However, the stability of the double assisted gas is impacted by the higher melt flow rate. When the melt flow rate increases to a certain high value, DL-GAE failure occurs. The DL-GAE of polymer micro-catheters fails because the higher melt flow rate produces a greater shear stress and N_1_ in the double assisted gas, causing the flow velocity and N_1_ for the double assisted gas to fluctuate; this destroys the stability of the double assisted gas.
(2) Regarding the influence of the double assisted gas, under a fixed melt flow rate, the pressure of the double assisted gas impacts the extrusion molding of polymer micro-catheters. With a synchronized increase in double assisted gas pressures, the diameters of the polymer micro-catheters increase, but their walls become progressively thinner. This occurs because the structure between the melt and double assisted gas is sandwich-like. The N_1_ of the double assisted gas exerted on the molten polymer increases with a synchronized increase in double assisted gas pressures. The diameters of the polymer micro-catheters increase, and their walls become thinner under the compression of double assisted gas with increased pressure.
(3) With an individual increase in the outer assisted gas pressure, surface bumps and corrugation defects are generated. The generation of surface bumps and corrugation defects on the outer surface of polymer micro-catheters may be induced by the greater N_1_ of outer assisted gas injected onto the outer surface of molten polymer. With an individual increase in the inner assisted gas pressure, the diameters of polymer micro-catheters increase rapidly until the inner cavity bursts. This occurs because the first normal stress of the inner assisted gas is injected onto the surface of the inner cavity of the polymer micro-catheters, and the inner assisted gas that cannot be discharged outside in time generates accumulating pressure. The polymer melt cannot withstand the high gas pressure in the inner cavity, causing the inner cavity to gradually expand until it finally bursts.
(4) To achieve DL-GAE molding of polymer micro-catheters in practice, the process parameters of the melt and double assisted gas should be controlled and maintained within a reasonable range. It is suggested that the parameters of the melt should match those of the double assisted gas. The pressures of the double assisted gas should not be excessive or deficient but should be reasonable and match the melt flow rate. When the flow rate of the melt increases, the pressures of the double assisted gas should also be increased.

## Figures and Tables

**Table 1:** Temperature controls of all sections for extruder barrel and DL-GAE die.

Sections	Extruder Barrel			DL-GAE Die	
	Section I	Section II	Section III	Section IV	Section V
*T* (°C)	200	210	215	220	220

**Table 2:** Material and rheological configurations of molten PP and double assisted gas.

Parameter	Molten PP	Gas
Total viscosity *η_k_* (Pa·s)	2700	2.6 × 10^−5^
Relaxation time *λ* (s)	0.2	0
*ε*	0.23	0
*ξ*	0.18	0
Viscosity ratio *η_r_*	0.12	0
Density *ρ* (kg/m^3^)	920	0.723
Specific heat capacity *C_pk_* (J/kg·K)	1883	1026
Thermal conductivity *k_k_* (W/m·K)	0.22	0.037
Viscous flow activation energy *E_γ_* (KJ/mol)	16,628	0

## Data Availability

The original contributions presented in this study are included in the article/Supplementary Material. Further inquiries can be directed to the corresponding authors.

## Supplementary Materials

The following supporting information can be downloaded at: https://www.mdpi.com/article/10.3390/polym17040504/s1, Figure S1: The photos of the assembled DL-GAE die and its components; Figure S2: The different morphologies of polymer micro-catheters for the synchronized increase pressure of double assisted gas via finite element numerical simulation.

## Abbreviations

DL-GAE	Double-layer gas-assisted extrusion
PTT	Phan–Thien–Tanner
PP	Polypropylene
GAE	Gas-assisted extrusion
