# Supplementary material for: Influences and Failure Analysis of the Interaction Between Melt and Gas on Double-Layer Gas-Assisted Extrusion Molding of Polymer Micro-Catheters
Source: Polymers (Basel). 2025 Feb 15;17(4):504. doi: 10.3390/polym17040504 (PMC11859098; doi:10.3390/polym17040504)
Supplement: Supplementary file 1 [file polymers-17-00504-s001.zip › polymers-3473114-supplementary.pdf]

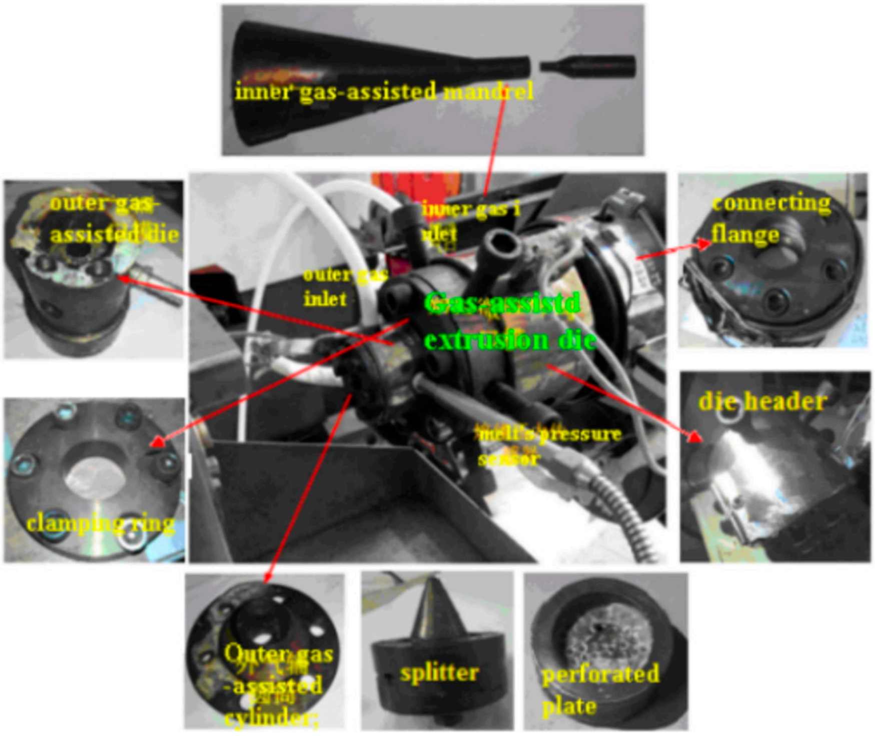

Figure S1. The photos of the assembled DL-GAE die and its components.

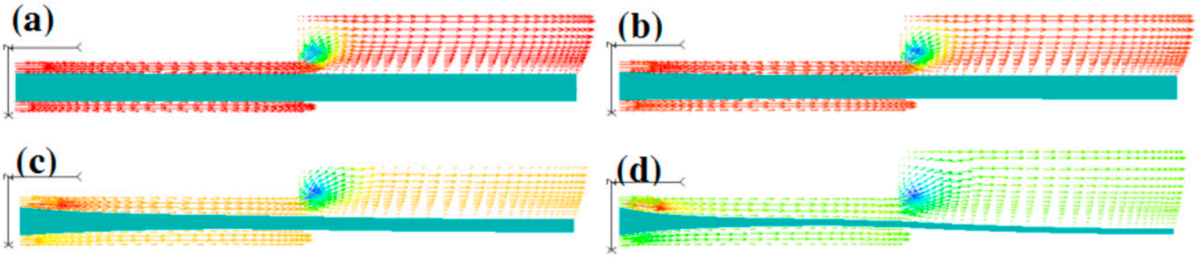

Figure S2. The different morphologies of polymer micro-catheters for the synchronized increase pressure of double assisted gas via finite element numerical simulation. (a) P_i=0.06 MPa, P_o=0.12 MPa; (b) P_i=0.065 MPa, P_o=0.13 MPa; (c) P_i=0.07 MPa, P_o=0.14 MPa; (d) P_i=0.075 MPa, P_o=0.15 MPa.
